# Increase in *Clostridium difficile*–related Mortality Rates, United States, 1999**–**2004

**DOI:** 10.3201/eid1309.061116

**Published:** 2007-09

**Authors:** Matthew D. Redelings, Frank Sorvillo, Laurene Mascola

**Affiliations:** * Los Angeles County Department of Public Health, Los Angeles, California, USA; † University of California Los Angeles–School of Public Health, Los Angeles, California, USA

**Keywords:** Clostridium difficile, mortality, death records, trends, vital statistics, dispatch

## Abstract

Reported mortality rates from *Clostridium difficile* disease in the United States increased from 5.7 per million population in 1999 to 23.7 per million in 2004. Increased rates may be due to emergence of a highly virulent strain of *C. difficile*. Rates were higher for whites than for other racial/ethnic groups.

*Clostridium difficile* is an anaerobic, gram-positive bacillus that can cause considerable disease, including diarrhea, colitis, and septicemia, resulting in death (*1*). *C. difficile–*associated disease (CDAD) primarily affects persons >65 years. Risk factors include residence in hospitals and long-term care facilities and the use of antimicrobial medications (*1*–*3*). Incidence of CDAD has been increasing, and severe cases are becoming more common (*4*,*5*). These changes in the incidence and severity of CDAD may be associated with the emergence of a more virulent strain of *C. difficile* bacteria (*5*,*6*). Death rates associated with *C. difficile* were reported to be increasing from 1999 to 2002 in the United States and from 2001 to 2005 in England and Wales (*7*,*8*). However, no trend analysis was conducted to evaluate the rate of increase. We incorporated mortality data for the United States through the year 2004 to conduct trend analyses of CDAD-related deaths and to examine demographic characteristics and coexisting conditions reported in deaths from *C. difficile* infection.

## The Study

CDAD*-*related deaths were identified by using multiple cause-of-death data from national mortality records for 1999–2004. CDAD*-*related deaths were defined as all deaths for which the underlying cause of death or any of the contributing causes of death included the International Classification of Diseases, 10th revision (ICD-10) code A04.7 (enterocolitis due to *C. difficile*). Information about the size and demographic breakdown of the US population for each year during 1999–2004 was obtained from censal and intercensal year estimates with bridged race data (*9*,*10*). Age-adjusted mortality rates were calculated by using the age distribution of the 2000 US population as a standard (*11*). The US population was divided into 5 racial/ethnic categories: white, Hispanic, Asian/Pacific Islander, black, and American Indian/Alaska Native.

During 1999–2004, CDAD was reported as a cause of death for 20,642 persons. CDAD was reported as the underlying cause for 12,264 (59%) of these deaths. A total of 3,256 deaths were reported related to all other intestinal infectious diseases combined (ICD-10 codes A00 to A09, excluding A047) over the same period. The median age of death for CDAD patients was 82 years. Age-adjusted mortality rates from CDAD were slightly higher for men than for women (Table) and were higher for whites than for any other racial/ethnic group. Most CDAD-related deaths occurred in hospitals (n = 16,557, 80%); 1,634 (8%) occurred in long-term care facilities, and 2,027 (10%) occurred at home.

**Table:** Demographic characteristics of patients with *Clostridium difficile*–related deaths, United States, 1999–2004

Demographic group	*C. difficile–*related deaths, no. (%)	Age-adjusted mortality rate/million population
Sex		
Female	12,468 (60)	11.8
Male	8,174 (40)	12.7
Race/ethnicity		
White	18,534 (90)	12.9
Hispanic	602 (3)	7.2
Black	1.304 (6)	9.3
Asian/Pacific Islander	139 (1)	3.5
American Indian/Alaska Native	63 (<1)	7.9
Age group, y		
<1	17 (<1)	0.7*
1–4	11 (<1)	0.1*
5–14	12 (<1)	0.1*
15–24	24 (<1)	0.1*
25–34	62 (<1)	0.3*
35–44	171 (1)	0.6*
45–54	464 (2)	2.0*
55–64	1,159 (6)	7.6*
65–74	3,238 (16)	29.3*
75–84	7,859 (38)	104.0*
>85	7,623 (37)	287.1*
Total	20,642	12.2

*This statistic is not age-adjusted because it only pertains to 1 age group.

Common coexisting conditions for CDAD-related deaths included septicemia (n = 7,654, 37%), renal failure (n = 4,786, 23%), pneumonia (n = 3,430, 17%), urinary tract infection (n = 1,496, 7%), and anemia (n = 785, 4%). HIV was reported for 81 CDAD-related deaths (<1%). However, among the 697 deaths reported in persons 25–54 years of age, HIV was reported for 72 (10%).

The overall rate of *C. difficile–*related deaths during the study period was 12.2 deaths per million population. Mortality rates related to CDAD increased during the study period (Figure), rising from 5.7 deaths per million population in 1999 to 23.7 deaths per million population in 2004. Poisson regression estimates showed mortality rates increased by 35% per year (coefficient = 0.30, standard error = 0.004, 95% confidence interval = 0.29–0.31) during the study period.

**Figure:**
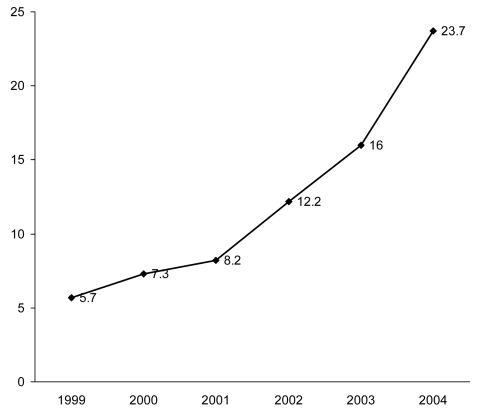
Yearly *Clostridium difficile–*related mortality rates per million population, United States, 1999–2004.

## Conclusions

Due to the inclusion of CDAD-related deaths when CDAD was not reported as the underlying cause of death, reported death rates in this study were higher than those published in an earlier analysis of CDAD-related deaths in the United States (*7*). *C. difficile* is a cause of a substantial and increasing proportion of deaths in the United States and may be underrecognized as a cause of death. Little attention has been paid to *C. difficile* prevention; media and public health awareness efforts have focused largely on the prevention of disease from other intestinal pathogens such as *Escherichia coli* or *Salmonella*. However, the incidence of deaths from *C. difficile* is greater than the extent of deaths from all other intestinal infectious diseases combined. *C. difficile*–related mortality rates were higher for whites than for other racial/ethnic groups. Racial/ethnic differences in insurance status and access to care (*12*) may render elderly whites more likely to receive treatment with antimicrobial drugs that put them at risk for *C. difficile* infection. However, genetic or other factors may also be involved, and further research is needed to determine the causes of racial/ethnic differences in *C. difficile*–related deaths.

Previous research showed increases in CDAD-related mortality rates in the United States until 2002 (*7*,*8*). This analysis estimates the rate of increase at 35% per year, and shows that mortality rates continued to increase at least until 2004. Increases in incidence and deaths from CDAD may be associated with the emergence of a new and more virulent strain of *C. difficile* (*5*). The emergence of virulent strains of *C. difficile* makes continued assessment of mortality statistics important.

Infection with *C. difficile* is associated with recent use of antimicrobial medications and with residence in hospitals. Most CDAD cases are acquired in healthcare settings (*1*), and as many as 90% of cases may be associated with antimicrobial drug use (*2*,*3*). High *C. difficile* death rates call attention to the importance of proper infection control practices in hospitals and long-term care facilities and the judicious use of antimicrobial medications. Further research is needed to explore current questions concerning which antimicrobial medications, if any, will lead to CDAD (*13*,*14*).

Infections such as septicemia, pneumonia, and urinary tract infections were commonly reported in conjunction with *C. difficile–*related deaths. For some of these patients, the administration of antimicrobial medications to treat infections from other pathogens may have paved the way for infection with *C. difficile.* However, other risk factors are known, so that in many cases the careful use of antimicrobial agents may not be enough to prevent *C. difficile* infection. HIV infection was only reported in a small fraction of CDAD*–*related deaths. However, immunosuppression and the use of prophylactic antimicrobial drugs in persons with AIDS may increase the risk for CDAD (*15*), and the effects of HIV should not be overlooked. In persons 25–54 years of age, in whom HIV infection is most common, HIV infection was reported in approximately one tenth of CDAD*-*related deaths. Thus, HIV can considerably increase *C. difficile* death rates for demographic groups in which HIV prevalence is high.

Death certificate data may underrepresent the extent of CDAD-related deaths. This analysis was limited to deaths in which ICD-10 code A047 (enterocolitis due to *C. difficile*) was mentioned and may have failed to capture CDAD-related deaths in which colitis was not present. In addition, death certificate data may be affected by reporting error. Supplemental information such as decedents’ medical histories was unavailable. No data were available regarding which strains of *C. difficile* were responsible for reported CDAD*–*related deaths.

*C. difficile* is an underrecognized cause of severe illness and death. This analysis underscores the importance of CDAD as a public health problem and the increasing incidence of CDAD-related deaths in the United States.
